# Benzoate of Mercury in Syphilis

**Published:** 1914-01

**Authors:** 


BENZOATE OF MERCURY IN SYPHILIS. G. B. Sweeney of Pittsburgh, *Urologic & Cutaneous Review,* having observed the results obtained by Gaucher of the St. Louis Hospital, Paris, who used a solution in sodium chlorid—sterile water, conceived the idea of eliminating the drawback of pain by employing an oil emulsion derived from bullock's brain. This medium and the precaution of using chemically pure benzoate of mercury proved successful in rendering hypodermatic injection comparatively painless. The patient may be assured that the period of treatment will seldom exceed three months; that there will be no digestive disturbances and that recurrence of lesions need not be feared if reasonable precautionary measures are followed up and correct habits are observed.

